# Angle at the Medial Border: The Spinovertebra Angle and Its Significance

**DOI:** 10.1155/2015/986029

**Published:** 2015-10-07

**Authors:** G. S. Oladipo, E. O. Aigbogun, G. L. Akani

**Affiliations:** Department of Anatomy, College of Health Sciences, University of Port Harcourt, Port Harcourt, Rivers State, Nigeria

## Abstract

*Background. *The evolution from quadrupedalism to bipedalism has adjusted the balance of the upper limb to extensive movement at the shoulder. The scapular angles provide the point of attachment and control to various muscles and have been associated with the different movements of the shoulder girdle and joint. This has made the morphometric and anthropometric study of scapula a subject of extensive investigation. *Aim.* In the present study, the angle at the medial border was measured in the South-Southern Nigerian population and an anatomical name was ascribed to the angle. *Method.* The study was conducted on 173 scapulae (75 right and 98 left) obtained from various Anatomy Department of South-Sothern Nigerian Universities. The angle at medial border was obtained by pinning the edge of the superior and inferior angles, the lined traced out, and the angle measured using a protractor. SPSS version 20 was used to analyse the data. *t*-test was used to determine mean angular difference in the sides. *Result.* The mean ± SD of the medial angle was observed to be 136.88 ± 7.70° (R = 138.13 ± 7.06° : L = 135.92 ± 8.05°). Statistical analysis using the *Z*-test for mean difference showed the medial angle was found to be higher in the right side of the scapula (mean difference of 2.214 ± 1.152°), but the observed difference was not statistically significant (*P* > 0.05). The above findings have adjusted the scapula from three to four angles (lateral, superior, inferior, and medial) formed from four borders (lateral, superior, inferior, and superomedial and inferomedial). The medial angle because of its anatomical location was named “*spinovertebral*” angle, owing to its position at the scapulae spine, and located in medial proximity to the vertebra column. *Conclusion.* The medial angle (now referred to as the spinovertebral angle) of the right side of the scapula is wider than the left. The representation of the spinovertebral angle is very important, as the directional attachment of the levator scapulae may be altered if it increases or decreases greatly hence resulting in stiffness of the neck. At this point, it could be postulated that the scapular is quadrangular rather than triangular.

## 1. Introduction

The scapulae are a pair of flat bones, approximately triangular in shape [1, 2]; situated dorsally on the rib cage, they lie between the second rib superiorly and the seventh to ninth rib inferiorly. Three borders enclose the body of the scapula: the superior, medial, and lateral borders. The superior border is the shortest and the sharpest of the three borders and it ends at the scapular notch [1]. The medial border, also called the vertebral border, is the longest of the three borders; it exhibits either a concave, convex, or straight pattern [1]; and it runs parallel to the vertebral column [3]. The lateral border also referred to as the axillary border is thick and runs inferiorly from the inferior edge of the glenoid cavity. The three borders meet strategically to form the referenced three angles (lateral, superior, and inferior) [2, 3].

The thin medial border of the scapula runs parallel to the spinous processes of the thoracic vertebrae; hence, it is often called the vertebral border [2]. Four muscles attaches to the medial border. At the full extent of the anterior lip is the insertion of serratus anterior, the posterior lip gives insertion to the levator scapulae (uppermost), rhomboid minor (middle), and the rhomboid major (lower middle) [2, 4, 5].

The scapula bears various angles, namely, superior, inferior, lateral [2, 3], medial, acromial, and coracoid angle [6]. The superomedial scapular angle which is difficult to measure has only been described in clearly pathological cases [7].

The present study investigates the anthropometry of the medial angle, possible anatomical name, and its significance to the muscular attachment along its borders. Externally, a normal scapula can be seen to have four angles (lateral, superior, inferior, and medial) formed from four borders (lateral, superior, inferior, and superomedial and inferomedial). The position of the medial angle is of significant importance in muscle biomechanics.

## 2. Methods

The material for this study was 173 scapulae comprising 75 (43.4%) right scapulae and 98 (56.6%) left scapulae of undetermined sex and age, obtained from Department of Human Anatomy of various South-Southern Universities in Nigeria. All the bones were well macerated and free from any physical deformity or abrasion and were complete in all respects.

The scapulae used in the present study were placed on a plane A4 paper held to the drawing board in a pronated position (i.e., the dorsal surface facing superiorly). The various points pined were the superior angle, inferior angle, and the sharpest edge at the origin or root of the scapula spine (Figures 2(a) and 2(b)). The medial angle (MA) was defined as follows.

**Figure 1:**
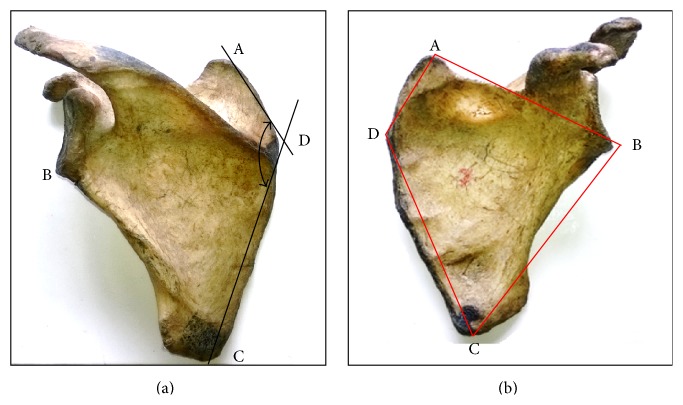
(a) The measured angles: SVA $\left(A\hat{D}C\right)$ and landmarks. (b) Borders of the scapula forming the various angles (line AB: superior border, line BC: lateral border, line AD: superior-medial border, line DC: inferior medial border).

**Figure 2:**
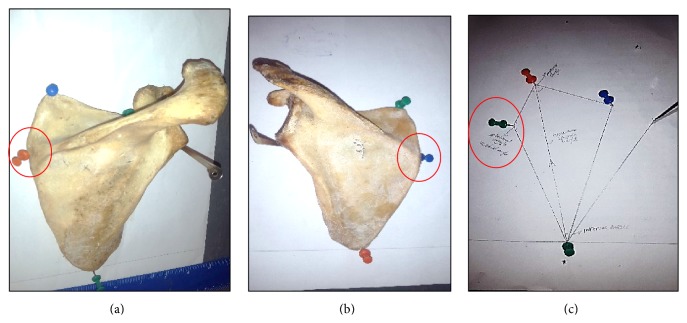
(a, b) Landmark and marking of the left and right medial scapula angle, respectively, (c) line drawn to mark out the observed medial angle.

*The Medial Angle (MA)*. The angle $\left(A\hat{D}C\right)$ is formed from the line (AD) joining the most superior point of the scapula to the most medial point of the medial border of the scapula (D), forming the superior-medial border of the scapulae and an adjoining line from the most inferior point of the scapular (B) to the most pointed edge of the medial border of the scapula (D) (Figures 1(a) and 1(b)).

Lines (A-D and C-D) were traced from the various pined points on the paper using a graphite HB pencil; the resultant angle formed was measured using a protractor (Figure 1(a)).

## 3. Results

The values observed were tabulated and the mean value and range were calculated for left and right side (Table 1). ANOVA and *t*-test were used to compare the mean difference in the values obtained for the sides (Table 2) and the comparison with other studies is documented in Table 3.

## 4. Discussion

The evolution from quadrupedalism to bipedalism has adjusted the balance of the upper limb to extensive movement at the shoulder. According to Bardin [8], developmentally, the scapula is triangular in nature; its angles in relation to muscle attachment are implicated in the wide range of movements at the shoulder joint [4, 8].

There have been various findings about the scapular morphology, anthropometry, kinanthropology, and kinematics. It is widely accepted that the scapula's shape, location, and muscular attachment greatly influence the mobility [9–12]. Several authors such as Larson and Stern [9], Larson [10], and Roberts [13] have discussed the role of forelimb function in dictating scapular shape. Inman et al. [14] highlighted some evolutionary changes of scapula from primates to human beings while Dwight [15] described the variations of the shoulder blade. Hamill and Knutzen [4] gave accounts of the biomechanical basis of human movement with reference to the shoulder girdle.

Sharma et al. [6] and Solanki [7] investigated the shape of the scapular and its size and movements in correlation with movements of the shoulder joint. In addition to range-of-motion at the shoulder, muscle function reportedly influences scapula form [13, 16, 17]. Codman and McLaughlin [18, 19] and Amasay and Karduna [20] briefly discussed the kinanthropology of the scapula. Anthropometric measurement of the scapulae has been extensively studied by Inman et al. [14] Dwight [15], and Graves [21] with sexual difference, height, and stature estimation reported [22–25].

However, all of them except Inman et al. [14] and recently Sharma et al. [6] recognized the anatomical significance of the different angles of the scapula in providing the base, direction, and leverage to various muscles and thus determining their effective roles in different movements.

*Medial Angle*. The mean value was found to be 136.88 ± 7.70° with a higher value for the right side (138.13 ± 7.06°) as compared with the left side (135.92 ± 8.05°) in the present study (Table 1), although the observed mean difference (2.214 ± 1.152°) was not statistically significant (*P* > 0.05) (Table 2). When compared with the two available studies by Solanki [7] and Sharma et al. [6], their values were higher than the values obtained for the Nigerian population (Table 3). The mean value of the medial angle obtained by Solanki [7] was comparable with that in the present study, whereas that of Sharma et al. [6] was about 10.76° wider than the mean value obtained in this study. This wide difference may have been as a result of methodological difference as Sharma et al. [6] measured the external borders; this present study considered the joining points of all angles (Figures 1(a) and 1(b)).

The angular description of the scapula has remained triangular even with the recent findings of Sharma et al. [6] where they described the medial angle of the scapulae with significant landmarks. The findings of this study have led us to believe that the scapula is quadrangular rather than triangular morphometrically and the inclusion of the medial border now regarded as the spinovertebral angle increases the external angle of the scapular to four (4) in contrast with the current documentation by Moore and Dalley [2], Marieb and Mallatt [3], Moore et al. [26], and other recognized scholarly publications.

### 4.1. Significance of the Spinovertebra Angle (Medial Angle)

The medial border of the scapula has always been regarded as a straight continuous border, but careful observation of all measured scapula indicates that the medial border of the scapula possesses two distinct areas (borders) for muscular attachment. Medial border from the pointed edge close to origin of the spine extending to the superior angle of the scapula can be regarded as the superomedial border which gives attachment to the levator scapulae. Another border from the origin of the scapula spine to the inferior angle of the scapula can be regarded as the inferomedial border which gives attachment to the rhomboid group of muscles (rhomboid minor, superiorly, and rhomboid major, inferiorly). The borders are illustrated in Figure 1(b).

**Figure 3:**
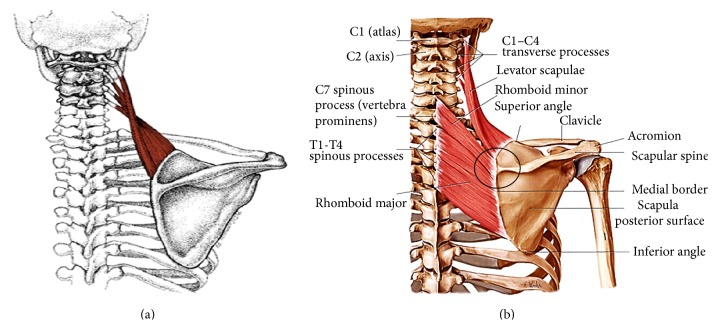
(a) Schematic illustration of the attachment of levator scapulae (http://www.crossfitsouthbay.com/muscle-spotlight-levator-scapula-2/) and (b) the levator scapula muscle and the rhomboid muscles (http://www.bestperformancegroup.com/wp-content/uploads/2014/03/serratus-anterior-rhomboids-and-levator-scapulae31).

The levator scapula originating from the first four cervical vertebrae (C1–C4) attaches to the superomedial border extending to the superior angle of the scapula in a downward diagonal manner; this is due to the position of the superomedial border of the scapula:

1. Alteration or widening of this border may increase the steepness of the muscle, altering the attachment of the fibres of the levator muscle. This may also create slacks within the lower fibres at the origin resulting in the inability of the levator muscle to pull the scapulae superomedially and possible loss of the burden bearing ability of the muscle when a heavy load is carried on the shoulder (Figure 3(a)).
2. The spinovertebra angle anatomically differentiates the inserting fibres of levator muscle and the rhomboid minor muscle. This structurally discriminates the function of the rhomboid muscle and the levator muscles. Attachment of fibres of the rhomboid muscle across the spinovertebra angle may give it an elevating function as well as its retracting action when trapezius is contracted (Figure 3(b)).

## 5. Conclusion

In summary, this research has provided baseline data for the medial angle of the scapulae for the Nigerian population. This research has postulated that medial angle may be referred to as the spinovertebra angle. It has also defined the role in providing base, direction, and leverage to various muscles. With the observation of superomedial and inferomedial borders which form the spinovertebrae angle, the scapular may now be structurally described as being quadrangular rather than a triangular bone.

## Figures and Tables

**Table 1:** Descriptive characteristics of the medial angle of the scapula.

	*N*	Mean ± SD	Std. error	Minimum	Maximum	Range
Right side (°)	75	138.13 ± 7.06	0.81569	123	157	34
Left side (°)	98	135.92 ± 8.05	0.81366	120	154	34
Total (°)	**173**	**136.88 ± 7.70**	**0.58532**	**120**	**157**	**37**

**Table 2:** Analysis of variance and mean difference in the sides of the medial angle.

Parameters compared	Test for equality of variances		*t*-test for equality of means				Inference
	*F*	Sig.	*t* (cal.)	*P* value	Mean difference	Std. error difference	
Medial scapula angle (°)							
Right side	3.825	0.052	1.96	0.056	2.214	1.152	NS
Left side							

**Table 3:** Comparison of the observed values of this study with earlier studies.

S/number	Parameters	Authorities	Scapula observed	R	L	Region	Mean ± SD
1	MSA	Present study	173 (R = 75, L = 98)	136.8°	135.9°	Nigerians	136.88 ± 7.7°
2	MSA	Solanki [7]		133°–173°	126°–170°	Indians	142.20°
3	MSA	Sharma et al. [6]	100 (R = 50, L = 50)	151.3°	143.96°	Indians	147.64°
